# Comprehensive Vaccine-Preventable Disease Surveillance in the Western Pacific Region: A Literature Review on Integration of Surveillance Functions, 2000–2021

**DOI:** 10.9745/GHSP-D-22-00017

**Published:** 2022-10-31

**Authors:** Morgane Donadel, Heather M. Scobie, Roberta Pastore, Varja Grabovac, Nyambat Batmunkh, Stephanie O’Connor, Benjamin A. Dahl, Christopher S. Murrill

**Affiliations:** a Global Immunization Division, U.S. Centers for Disease Control and Prevention, Atlanta, GA, USA.; b World Health Organization, Western Pacific Regional Office, Manila, the Philippines.

## Abstract

**Introduction:**

A strategic framework for 2021–2030 developed by the World Health Organization (WHO) Regional Office for the Western Pacific emphasizes the need for high-quality and integrated vaccine-preventable disease (VPD) surveillance. We conducted a literature review to document the barriers, enabling factors, and innovations for integrating surveillance functions for VPDs and other communicable diseases in Western Pacific Region (WPR) countries.

**Methods:**

We searched published and gray literature on integrated VPD surveillance from 2000 to 2021. Articles in English, Spanish, or French were screened to identify those relating to VPD surveillance in a WPR country and not meeting defined exclusion criteria. We categorized articles using the 8 WHO surveillance support functions and abstracted data on the country; type of surveillance; and reported barriers, enabling factors, and best practices for integration.

**Results:**

Of the 3,137 references screened, 87 met the eligibility criteria. Of the 8 surveillance support functions, the proportion of references that reported integration related to the laboratory was 56%, followed by workforce capacity (54%), governance (51%), data management and use (47%), field logistics and communication (47%), coordination (15%), program management (13%), and supervision (9%). Several references noted fragmented systems and a lack of coordination between units as barriers to integration, highlighting the importance of engagement across public health units and between the public and private sectors. The literature also indicated a need for interoperable information systems and revealed the use of promising new technologies for data reporting and laboratory testing. In some WPR countries, workforce capacity was strengthened at all administrative levels by the implementation of integrated trainings on data monitoring and use and on laboratory techniques applicable to multiple VPDs.

**Conclusion:**

This literature review supports integrating VPDs into broader communicable disease surveillance systems in WPR countries while ensuring that the minimal WHO-recommended standards for VPD surveillance are met.

## Abstract (precis)

A literature review supports integrating vaccine-preventable diseases (VPDs) into broader communicable disease surveillance systems in Western Pacific Region countries while ensuring that the minimal World Health Organization–recommended standards for VPD surveillance are met.

## Teaser

**Key Findings:**

- We document factors that influenced integrating surveillance functions for vaccine-preventable diseases (VPDs) and other communicable diseases in the Western Pacific Region (WPR).
- Barriers included insufficient coordination within the public and private sectors, inadequate engagement of national Expanded Program on Immunization programs, lack of surveillance and laboratory capacity, inability to link epidemiologic and laboratory data, and suboptimal scope and design of surveillance systems for achieving control and elimination goals.
- Best practices and innovations included developing guidelines for integrated VPD surveillance, standardizing processes for laboratory surveillance and testing of multiple VPDs, using multiplex testing for multiple diseases, and conducting joint epidemiology and laboratory surveillance data review meetings.

**Key Implications:**

- National WPR stakeholders should consider (1) using the outcomes of this review to inform country implementation plans and (2) developing legal frameworks, guidance documents, and coordination mechanisms for reporting and investigation of cases and clusters of VPDs and other non-VPDs of public health importance.
- Public health program managers should consider opportunities for surveillance integration, such as in workforce and laboratory capacity, and information systems and data management.

## INTRODUCTION

The status and quality of vaccine-preventable disease (VPD) surveillance vary by disease and country. Globally, most countries have national case-based surveillance for polio, measles, and neonatal tetanus. Some countries may also have sentinel site case-based surveillance for 1 or more of these diseases. For both types of disease-specific, case-based surveillance systems, standard components of surveillance include detection, notification, investigation, and laboratory confirmation of suspected cases, within a defined surveillance area. In parallel, countries may also have aggregate weekly reporting of nationally notifiable diseases, and in some cases, event-based surveillance to capture reports from the community and media.^1^ Detected VPD outbreaks may be investigated; however, reporting often only consists of aggregate data with limited laboratory confirmation, which may be of limited use for decision making by immunization programs.^2^^,^^3^

Recently, national governments adopted the World Health Organization (WHO) Immunization Agenda 2021–2030, which includes as a core principle using reliable and timely data to track progress, drive improvements in performance, and underpin decision making at all levels of the program.^4^ A companion document to the Immunization Agenda 2030 is the Global Strategy on Comprehensive Vaccine-Preventable Disease Surveillance,^2^ which aims to secure and accelerate the development of country-led and country-owned comprehensive VPD surveillance systems, including integration of the WHO-defined support functions (governance, program management, workforce capacity, laboratory, field logistics and communication, supervision, data management and use, and coordination) and resourcing across diseases.^2^ This strategy provides a framework to bring together different types of surveillance for both viral and bacterial pathogens while highlighting the critical components of VPD surveillance needed to generate data to drive decision making and policy. Similar to the global strategy, the Regional Strategic Framework for Vaccine-Preventable Diseases and Immunization in the Western Pacific 2021–2030,^5^ which was developed by the WHO Regional Office for the Western Pacific (WPRO) and endorsed by the Regional Committee in October 2020, emphasizes the need for high-quality VPD surveillance with integrated functions.

Many countries in the Western Pacific Region (WPR) conduct VPD surveillance using both disease-specific and integrated systems. A prominent example of the latter are the early warning and response systems originally designed for outbreak detection in emergency settings,^6^ which have been adapted as long-term national routine surveillance systems used for VPD surveillance in Cambodia, Lao People's Democratic Republic (PDR), and Mongolia. Analysis of data from the 2017 Joint Reporting Form supplemental questionnaire on VPD surveillance in the WPR showed that 10 (59%) of the 17 countries or areas that reported data have an integrated surveillance system for all or most VPDs.^7^

Many WPR countries conduct VPD surveillance using both disease-specific and integrated systems.

To support the implementation of the regional strategic framework, we conducted a literature review to document the barriers, enabling factors, and best practices including innovations for integrating surveillance functions for different VPDs and other communicable diseases in WPR countries.

## METHODS

### Search Methods

Following the Preferred Reporting Items for Systematic Reviews and Meta-Analyses guidelines,^8^ 2 complementary search strategies were developed and used in 7 databases (Medline, Embase, Global Health, CINAHL, Scopus, ProQuest Central, and Africa Wide Information) (Supplement Table S1). The first search included terms combined with Boolean operators AND and OR relating to “vaccine preventable disease” or “vaccine preventable infection” or “vaccine preventable illness” and “surveillance” and “integrate” or “expand” or “comprehensive” or “link.” The second search included search terms relating to “syndromic surveillance” or “disease surveillance” or “outbreak surveillance.” Both searches were limited to the years 2000–2021 and conducted only in English. Additionally, key VPD surveillance staff from the WPRO, the WHO Headquarters, and the U.S. Centers for Disease Control and Prevention were consulted to provide any references relevant to this topic. Gray literature was included in the review using the definition by the Cochrane Handbook for Systematic Reviews of Interven-tions.^9^ Such literature was referred by key informants. It included reports and presentations from VPD surveillance reviews, Expanded Program on Immunization (EPI) reviews, Regional Technical Advisory Group on Immunization and VPDs meetings, Regional Verification Commission for Measles and Rubella Elimination meetings, Regional Certification Commission for Poliomyelitis Eradication meetings, Regional VPD Laboratory Network meetings, and country support missions.

### Data Collection

Two reviewers independently screened titles and abstracts from the references, assessed the abstracts and the full-text articles for eligibility, and abstracted articles of the first literature search from 2000 to 2019; 1 reviewer screened and abstracted the first literature search references from 2019 to 2021 and the whole of the second literature search. The following exclusion criteria were used: (1) did not include individual or multicountry experience from any country of the WPR; (2) did not focus on human disease (e.g., focused on animal disease); (3) did not include VPD surveillance, where VPDs are defined as cholera, congenital rubella syndrome, diphtheria, *Haemophilus influenzae* type b, hepatitis A, hepatitis B, human papillomavirus, influenza, Japanese encephalitis, measles, meningococcus, mumps, tetanus, pertussis, pneumococcus, polio, rotavirus, rubella, typhoid, varicella, and yellow fever; (4) consisted only of an analytical study (e.g., estimating the burden of disease), not describing the surveillance system; (5) focused only on surveillance of adverse events following immunization; (6) language of the article other than English, Spanish, or French. We used the definition of integration provided by the Global Strategy on Comprehensive Vaccine-Preventable Disease Surveillance to determine the eligibility of references.^2^ As such, we included references that described integration of VPD surveillance systems with each other or into other existing VPDs and other non-VPDs’ surveillance systems through the WHO-defined surveillance support functions. Any discrepancy between reviewers in the processes of screening was discussed and resolved through consensus.

We categorized the areas of reported integration using the WHO-defined VPD surveillance support functions (Table 1).^2^ For example, we categorized integrated guidelines as integrated governance. We also abstracted data on the country, the country income level, the VPD or other communicable disease of interest, the type of surveillance (e.g., aggregate or case-based), the level of implementation (i.e., national or subnational), and the context for integration (i.e., routine program, study/research, or outbreak response). Country income levels were defined using the 2019 World Bank definitions, based on country gross national income per capita and the World Bank Atlas method and countries were classified as low income, lower-middle income, upper-middle income, or high income.^10^ For each WHO-defined surveillance function, we abstracted text describing any barriers, enabling factors, and best practices with surveillance integration. When reported, the effectiveness of integration on surveillance operations was abstracted. Data abstraction was conducted using Covidence, an online systematic review management program. The synthesis of the evidence used both narrative and tabular formats and characterizes the quantity and quality of the literature.

**TABLE 1.** Potential Areas of Integration Among Vaccine-Preventable Disease Surveillance Support Functions^a^

**Surveillance Support Functions**	**Potential Areas for Integration**
Governance	Standards and guidelines development, policy, laws/mandates, roles and responsibilities (including for private sector), and funding
Program management	Budget creation, resource mobilization, financial management, sustainability, infrastructure/equipment management, human resources, and external surveillance assessments and reviews
Workforce capacity	Training/capacity building at all levels; staff for core functions including case detection, notification, investigation, reporting, and response; and epidemic preparedness
Laboratory	Specimen collection kits, reagents, and supplies; equipment; physical space; training; personnel; expansion and diversification of regional and global networks; shared procurement processes; and quality management systems
Field logistics and communication	Airtime and Internet for notification and reporting, specimen collection, and transport; and feedback of results
Supervision	Supportive supervisory visits, workplans, and checklists
Data management and use	Information system development; and data harmonization, implementation, and use for performance improvement
Coordination	Linking surveillance program to relevant stakeholders (e.g., EPI) for data review, dissemination, and use; improvement planning; surveillance strengthening as core function of International Health Regulations implementation framework, including rapid response teams and emergency operations centers

Abbreviation: EPI, Expanded Program on Immunization.

a From the World Health Organization Global Strategy on Comprehensive Vaccine-Preventable Disease Surveillance.^2^

We categorized the areas of reported integration using the WHO-defined VPD surveillance support functions.

## RESULTS

The searches identified a total of 4,471 references from the first search and 680 references from the second search (Figure). After removing the 2,014 duplicates, we screened 3,137 references; 1,800 were excluded based on the exclusion criteria. This left 1,337 full-text references and 42 gray literature references to be assessed for eligibility using the exclusion criteria. After removing 74 duplicates and excluding 1,218 articles based on the exclusion criteria, we were left with a total of 87 references.^7^^,^^11^^–^^96^ Almost half (48%) were gray literature references. Sixty-eight (78%) references reported data exclusively from countries of the WPR, while the remaining (22%) compiled data from countries located in the WPR and in other regions. Of the 87 references, 6 (7%) provided data from high-income countries, 11 (13%) from upper-middle-income countries, 34 (39%) from lower-middle-income countries, and none reported on low-income countries. The remaining 36 (41%) articles focused on a mix of economic levels while providing a regional or global perspective. All WPR countries were specifically referenced at least once in the articles included; experiences from China (16 references), Vietnam (14), Cambodia (13), Lao PDR (13), and the Philippines (12) were most frequently reported followed by Fiji (9), Mongolia (9), and Papua New Guinea (PNG) (9) (Supplement Table S2). In terms of the type of surveillance described, 66 (76%) of the 87 references reported on integrated systems that included case-based surveillance while the rest included aggregate or event-based surveillance. Supplement Table S3 summarizes the 87 included references.

**FIGURE.**
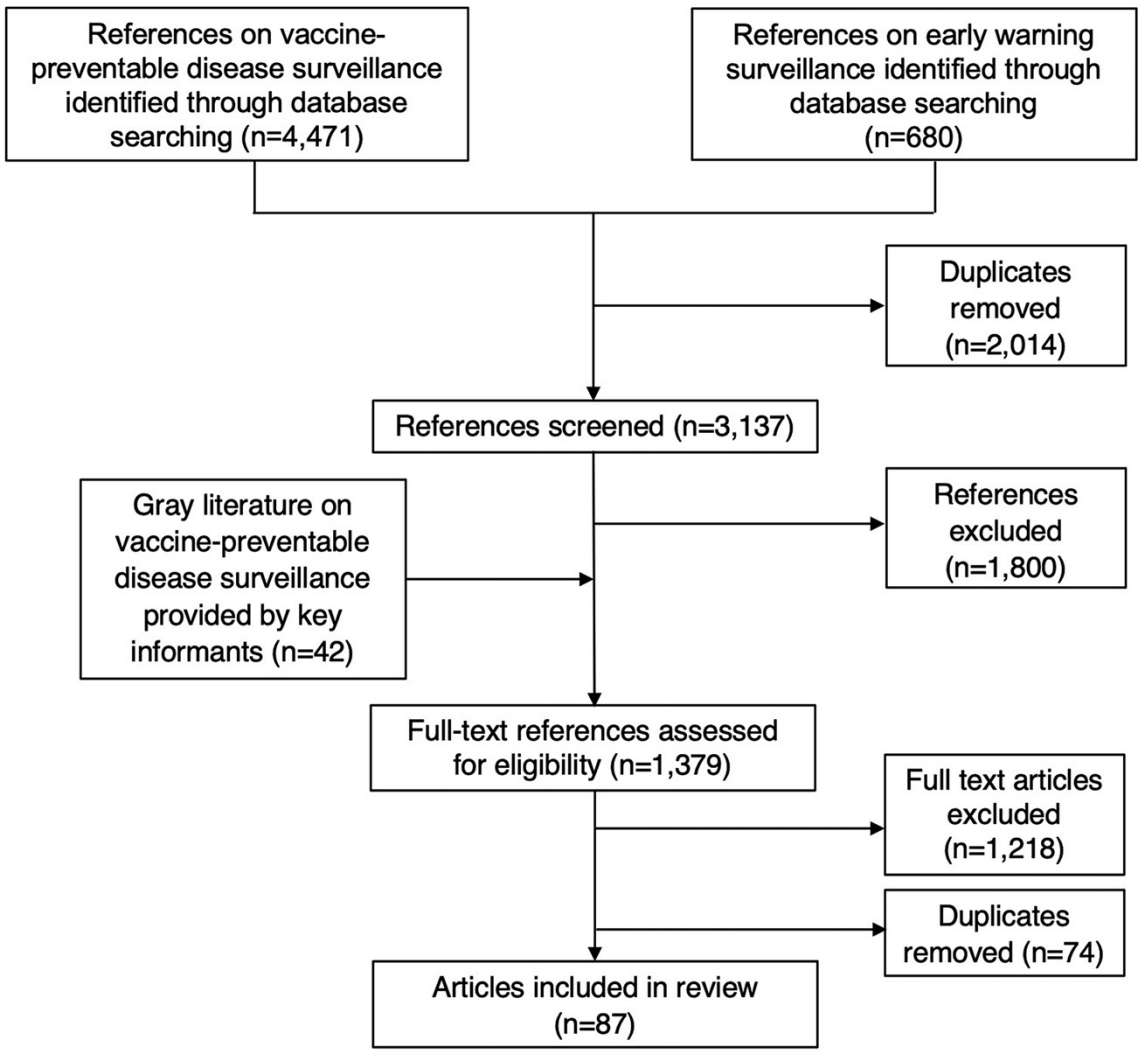
Flow Chart of Literature Review on Comprehensive Vaccine-Preventable Disease Surveillance in the WHO Western Pacific Region, 2000–2021 Abbreviation: WHO, World Health Organization.

Of the 8 surveillance support functions, the areas of reported integration related to laboratory (56%), followed by workforce capacity (54%), governance (51%), data management and use (47%), field logistics and communication (47%), coordination (15%), program management (13%), and supervision (9%) (Table 2). We review key challenges and best practices with each function in more detail.

**TABLE 2.** References Reporting Integration by WHO Surveillance Support Function

**Surveillance Support Functions**	**Published References, No. (%) (n=45)**	**Gray Literature References, No. (%) (n=42)**	**Total References, No. (%) (n=87)**
Laboratory	26 (58)	23 (55)	49 (56)
Workforce capacity	29 (64)	18 (43)	47 (54)
Governance	26 (58)	18 (43)	44 (51)
Data management and use	24 (53)	17 (40)	41 (47)
Field logistics and communication	20 (44)	21 (50)	41 (47)
Coordination	8 (18)	5 (12)	13 (15)
Program management	4 (9)	7 (17)	11 (13)
Supervision	2 (4)	6 (14)	8 (9)

Abbreviation: WHO, World Health Organization.

### Laboratory

Laboratory confirmation is an important complement to epidemiological surveillance, especially in elimination settings, where confirmation of suspected syndromic cases is needed, as well as molecular typing of VPDs in every suspected case.^59^ National integrated VPD surveillance systems with laboratory support exist in WPR countries for febrile rash illnesses (e.g., measles, rubella, dengue), diarrheal diseases (e.g., rotavirus), arboviruses (e.g., Japanese encephalitis and Zika), and bacterial VPDs (e.g., diphtheria and pertussis).^59^ However, issues with domestic public health laboratory capacity were reported in several WPR countries and areas such as the Pacific islands, inhibiting timely outbreak detection.^18^ Countries have progressively built their laboratory capacity by using their national laboratory networks, if any, and leveraging the WHO-coordinated networks (Table 3). These networks provide a framework for establishing quality standards, as well as resources for support and incentives to maintain those standards.

**TABLE 3.** Best Practices and Innovations in Comprehensive Vaccine-Preventable Disease Surveillance Implementation in the WHO Western Pacific Region, by WHO Surveillance Function

**Surveillance Support Functions**	**Best Practices and Innovations**
Laboratory	Using the same equipment, reagents, and supplies for testing multiple VPDs Integrating trainings on laboratory techniques applicable to multiple VPDs (e.g., bacterial VPD confirmation) Using multiplex tests or multiple tests of the same syndromic sample
Workforce capacity	Preservice and on-the-job competency-based training programs to strengthen surveillance data capacity at all levels of the surveillance workforce Adequate number of trained and experienced staff to conduct case detection, notification, investigation, reporting, and response
Governance	Engagement of national EPI in the process of development and implementation of integrated surveillance Government leadership in fostering collaborative partnerships between the public and private sectors National funding for integrated surveillance operations Quality operational guidelines for surveillance of multiple VPDs
Data management and use	Integrated/interoperable information systems, including different VPDs based on EPI user requirements and local context Linkage between epidemiological and laboratory surveillance data for individual cases Joint epidemiological and laboratory surveillance data review meetings Triangulation of coverage and VPD surveillance data to better identify immunity gaps in the population Surveillance data use for EPI planning Integrated VPD serosurveys and data use for EPI planning
Field logistics and communication	Using the same shipment mechanisms for specimen transport for multiple VPDs Using information and communication technology tools for clinician sensitization and notification of suspect cases to strengthen the representativeness and timeliness of reporting
Coordination	Linking surveillance to EPI program units for data review, dissemination, and use Emergency Operations Center’s coordination of surveillance, outbreak investigation, and response
Program management	VPD surveillance reviews, including multiple VPDs VPD surveillance workforce needs are addressed at all levels as part of the national human resource planning
Supervision	Regular supportive supervisory visits provided to surveillance staff of all administrative levels

Abbreviations: EPI, Expanded Program on Immunization; VPD, vaccine-preventable disease; WHO, World Health Organization.

Issues with domestic public health laboratory capacity were reported in several countries, inhibiting timely outbreak detection.

Due to demand, over the last decade, the laboratory networks in the region, the WPRO, and other partners, such as Japan International Cooperation Agency, have implemented integrated trainings on laboratory techniques (both serological and molecular methods) applicable to multiple VPDs, while strengthening quality assurance of laboratories.^36^^,^^59^ Another best practice of integrated laboratory management is the use of the same equipment, reagents, and supplies for testing multiple VPDs. In the Philippines, several programs (i.e., polio, measles, rubella, rotavirus, and Japanese encephalitis) are part of a national virology laboratory and share the same laboratory infrastructure, equipment, and some staff.^51^ WPRO staff observed that this integration leveraged capacity to maximize testing for accurate and timely detection and confirmation of VPD cases.

The use of multiplex tests on the same sample makes widespread laboratory confirmation of VPDs a more attainable goal while facilitating integration of syndromic surveillance.^86^^,^^88^^,^^96^ The U.S. Centers for Disease Control and Prevention recommends performing multipathogen diagnosis on diseases with severe outcomes, which increases the number of diseases diagnosed and reduces costs, both of which are better for low- and middle-income countries.^97^ The use of multiplex assays was considered across multiple pathogens when establishing Vietnam’s plan for nationwide serosurveillance.^87^ Invasive bacterial vaccine-preventable diseases sentinel surveillance systems allow the detection of multiple invasive bacterial pathogens using the same syndromic sample.^21^^,^^30^ Integrating Japanese encephalitis and dengue into Cambodia’s routine meningoencephalitis surveillance system enabled better estimation of its burden.^21^^,^^24^

A pilot study of measles rapid diagnostic test use as part of the Malaysia surveillance program showed promising results for timely public health response while enabling virologic surveillance.^98^

### Workforce Capacity

Public health surveillance can be a challenge in underresourced and understaffed settings. The high turnover of surveillance staff was reported as a major issue in operationalizing integrated surveillance that includes VPDs in Cambodia,^23^ Lao PDR,^45^ Mongolia,^49^ the Pacific islands,^11^ the Philippines, and Vietnam.^82^ This threatened consistency in adherence to VPD surveillance guidelines and created a need for frequent refresher trainings. The high turnover of laboratory staff limits the capacity to run tests as observed with the acute meningitis-encephalitis syndrome surveillance system in 2015 in the Philippines.^50^ Community-based programs that employ volunteers may lessen the burden on hospital workers in rapid outbreak detection, monitoring of communicable disease, and notification of vital events, such as noted in Cambodia and Lao PDR.^13^ However, beyond making VPD surveillance guidelines available, preservice and on-the-job trainings of the surveillance workforce are key to ensuring their adequate implementation during both routine operations and in the event of an outbreak (Table 3). If not, this can have detrimental effects on surveillance quality.

Preservice and on-the-job trainings of the surveillance workforce are key to ensuring guidelines are adequately implemented.

Inadequate knowledge among personnel in the Philippines, Vietnam, Malaysia, and China complicated reporting mechanisms, contributing to the underreporting of cases.^85^ Limited capacity for investigation, verification, and response activities was reported at the subnational levels in the Pacific islands,^18^^,^^78^ and in Lao PDR.^44^^,^^45^ Training on case detection, notification, investigation, data analysis, and response was deemed lacking in several countries including Lao PDR, Mongolia, Vietnam, and the Solomon Islands.^11^^,^^12^^,^^17^^,^^43^^,^^44^^,^^80^

Implemented in Lao PDR, the global data analysis and use training program called Stop Transmission of Polio Immunization and Surveillance Data Specialists, enabled building capacity on the management, analysis, and use of immunization and VPD surveillance data among the workforce at the subnational levels.^41^ In addition, trainings organized by the WHO WPRO have been progressively more integrated across VPDs, emphasizing cross-cutting competencies, namely immunization and VPD surveillance data management, analysis, and use for tailored action, as conducted in Vietnam and Lao PDR.^41^

### Governance

Different models of organizing and managing VPD surveillance impact its functionality (Table 3). In cases where ministry of health (MOH) surveillance units are responsible for managing VPD surveillance, potential challenges include poor communication and collaboration between the surveillance unit and the EPI; VPD information systems designed without input from the EPI and not collecting the required data elements for effective program management; irregular provision of VPD surveillance data to the EPI; and delayed or lack of coordinated outbreak vaccination response.^14^^,^^24^^,^^25^^,^^44^ In cases where the EPI manages VPD surveillance, the data collected usually provide information relevant for program planning; however, other challenges may occur, such as different surveillance guidance documents that may not be coordinated (e.g., different case definitions for the same syndrome or different approaches regarding the need to investigate and obtain laboratory confirmation of VPD); parallel surveillance reporting processes that may not be linked in terms of investigating VPD alerts; suboptimal surveillance capacity at the operational level, creating higher management demand for the national and provincial staff; and large discrepancies in the number of suspected cases of the same disease reported through the integrated and VPD surveillance systems.^22^^,^^23^^,^^80^

A challenge in multiple countries of the region is the insufficient role of the private sector in disease surveillance. The role of private practitioners in routine disease surveillance was found to be low in the Philippines, Vietnam, Malaysia, China, and Mongolia.^12^^,^^80^^,^^85^ Governments need to take leadership and foster collaborative partnerships between the public and private sectors and exercise regulatory authority where needed.^85^

Barriers to integration include parallel guidelines or a lack of guidance for surveillance and outbreak response. Reviews of VPD surveillance systems in Cambodia, Lao PDR, and Vietnam highlighted the need to develop VPD-specific guidelines to improve the quality of VPD case-based surveillance.^23^^,^^44^^,^^80^ In Tuvalu, public health staff noted quicker reporting and swifter and more assertive response measures during 2 typhoid outbreaks that they attributed to the new guidance for outbreak response.^78^

Engagement with key political partners is needed to ensure that any new surveillance systems do not conflict with existing priorities and that the systems are country-owned.^14^^,^^31^ After beginning as a pilot project in collaboration with external partners, management of funding and operations of China’s acute meningitis-encephalitis syndrome surveillance system were transferred under the National Health Commission.^26^^,^^27^ National investment in integrated surveillance was reported as a critical factor to the success of the implementation of the laboratory influenza network in China.^86^

### Data Management and Use

Integrated information systems including different VPDs should be designed based on EPI user requirements and local context (Table 3). The use of a unified reporting system was considered a critical enabling factor to the integration of acute meningitis-encephalitis syndrome surveillance with components of polio and measles infrastructure already in place in China.^27^ The existence of multiple surveillance reporting systems operating in parallel can lead to double- or triple-entry of case information, as reported in Mongolia.^49^ The potential flexibility of information systems to integrate data for other diseases in the future is another key factor to consider in the system design, as reported in the Philippines.^52^ Scaled up to support increased information demands, the Solomon Islands’ syndrome-based surveillance system enabled the MOH to monitor the evolution of a dengue outbreak and other febrile syndromes including measles.^11^ Another logistical issue observed in Lao PDR^45^ and in the Philippines^51^^,^^52^ is the lack of linkage between epidemiological and laboratory data within the same record. Of note, the WPRO supports the development and implementation of web-based information systems with linkage capacity for measles and rubella, rotavirus, acute flaccid paralysis/polio, and invasive bacterial vaccine-preventable diseases, and these systems are currently in use in several countries of the region.^69^

Examples of surveillance data use for program decision making include the Solomon Islands’ target response to the 2016 dengue outbreak.^11^ During the 2016 and 2018 measles outbreak responses in Cambodia, district-level risk assessments were conducted in areas with VPD cases to target supplementary immunization activities.^99^ In Mongolia, following the 2015 measles outbreak, an integrated VPD serosurvey was conducted and the data were used to fill immunity gaps.^99^

### Field Logistics and Communication

Logistical barriers to surveillance implementation were reported as inhibiting factors to timely outbreak detection in several countries/areas, including the Pacific islands.^18^^,^^78^ They include challenges with sample transportation to the national public health laboratory, especially in the remote districts of Mongolia^47^^,^^49^ or the small islands of the Philippines.^51^ These issues result in delays in specimen testing and variable performance of suspected cases with adequate laboratory confirmation. As with any new surveillance effort, a new syndromic surveillance system should, to the extent possible, be integrated into existing reporting pathways and build on existing public health surveillance infrastructure. The use of the same hardware, software, and communication infrastructure for timely outbreak detection can strengthen data collection, reporting, and interpretation for multiple public health programs. In China, routine surveillance for infectious diseases is done using an online system that allows for real-time, case-based reporting for nationally notifiable diseases.^55^ The use of an Internet-based disease reporting system for national molecular typing data of all bacterial infectious diseases allowed public health officials to identify disease outbreaks and implement timely responses.^16^ With increasing access to the Internet and decreased cost of information technology in developing countries, novel applications for syndromic surveillance represent opportunities to enhance surveillance and detection of outbreaks worldwide.^15^ Use of information and communication technology tools, such as short message service and the offline-capable Open Data Kit software suite, has strengthened the representativeness and timeliness of reporting of acute flaccid paralysis and syndromic surveillance in PNG.^100^^–^^102^

A new syndromic surveillance system should be integrated into existing reporting pathways and build on existing infrastructure.

Promoting timely data analysis and sharing of information through bulletins or reports targeting different audiences can help strengthen surveillance systems, data collection, and analysis.^86^ In Japan, effective and timely feedback of information to public health staff and the general public is prioritized through weekly, monthly, and ad-hoc infectious disease reports and journal publications.^83^

### Coordination

The lack of coordination between the surveillance units that include VPDs can create redundancy and overlap.^13^ Linking the VPD surveillance program to relevant stakeholders such as the EPI program when they are separate is critical as well as in the case of a decentralized MOH, where funding and administration are shared between national and local levels (Table 3).^17^

In many WPR countries, surveillance, outbreak investigation, and response are coordinated through an emergency operations center. Comprising senior-level MOH staff, provincial health authorities, local nongovernmental organizations, and development partners, the emergency operations center is instrumental to the country’s capacity to manage outbreaks.^11^ The establishment of provincial emergency operations centers in PNG enabled an unprecedented coordination to successfully respond to the circulating vaccine-derived poliovirus type 1 polio outbreak in 2018–2019.^57^

### Program Management

A reported barrier to integration is the lack of adequate domestic funding to sustain high-quality surveillance systems.^65^ In the Pacific islands, issues inhibiting timely outbreak detection included the lack of infrastructure for surveillance.^18^ Many operational concerns related to surveillance in PNG and in the Pacific islands are linked to challenges with sustainability, human resources, and finances, including operational funding for outbreak investigation and response.^18^^,^^55^^,^^56^

To continue efforts toward strengthening VPD surveillance systems, governments of Lao PDR, the Philippines, Vietnam, Mongolia, Cambodia, Malaysia, Fiji, and PNG have requested external VPD surveillance assessments and reviews.^65^ These were conducted, either through comprehensive VPD surveillance reviews, as part of comprehensive EPI reviews, or assessments of new vaccine surveillance.^65^

### Supervision

Ongoing supervision was recommended to strengthen the surveillance workforce capacity in several countries including Lao PDR and Vietnam.^17^^,^^44^^,^^85^ Conducted by surveillance focal points at each administrative level, with staff from higher levels visiting staff at lower levels, the supervisory visits are intended to identify and correct problems and provide technical assistance, mentoring, and hands-on refresher training as needed.^17^ Being an integral part of routine surveillance systems, supervisory visits should be integrated across diseases and surveillance programs to conserve resources.

## DISCUSSION

Significant progress has been made toward the quality and sustainability of VPD surveillance systems in WPR countries, especially for diseases with eradication and elimination goals. Fifty percent of WPR countries have included all or some VPDs in an integrated surveillance system.^62^ National integrated VPD surveillance systems with laboratory support exist for febrile rash illnesses, diarrheal diseases, arboviruses, and bacterial VPDs.^59^ However, there is large variability of VPD surveillance maturity and performance across countries.^62^ In some WPR countries, VPD surveillance systems are parallel, duplicated, or fragmented.^62^ The important investment made in surveillance systems for diseases targeted by elimination and eradication goals contributes to this situation. Integrated surveillance systems are more sustainable to maintain by countries, but challenges exist with ensuring these systems meet VPD-specific requirements and standards. Some countries have a suboptimal surveillance system scope and design, in terms of VPDs under surveillance, standard case definitions, and network of reporting facilities (e.g., excluding the private sector). There are many components of a surveillance system that can be integrated, thereby improving efficiency, and optimizing limited resources (e.g., streamlining reporting processes to facilitate case notification). Any approach to integrated VPD surveillance must be flexible and able to respond to local conditions^84^ (e.g., enabling active surveillance or line listing cases).

Any approach to integrated VPD surveillance must be flexible and able to respond to local conditions.

The literature review identified regional best practices, innovations, and pervasive challenges with laboratory, workforce capacity, governance, data management and use, and field logistics and communication. Issues related to coordination, program management, and supervision were reported to a lesser extent. Reported challenges in surveillance illustrate the need for improving efficiencies in resource utilization and strengthening integration of surveillance support functions at every level.

Improving laboratory capacity through standardized test procedures, quality control, and integrated trainings is critical for successful implementation of an integrated system. The increasing availability of multiplex and rapid diagnostic testing kits offers the potential to address testing capacity constraints in limited-resource and remote settings.^18^ Where feasible, efforts should be made to expand the capacity and implement new technologies for rapid detection and characterization of pathogens while creating efficient use of laboratory resources.

Reporting facilities and their staff are essential to any surveillance system. VPD surveillance workforce needs should be addressed at all administrative levels as part of national human resource planning. Implementing surveillance systems successfully requires a workforce adequately trained on case definition, detection, notification, and reporting. A systematic review by Rowe et al. highlighted that additional improvements in health care provider performance and the quality of health care in low- and middle-income countries occur when trainings are combined with other components, such as supervision, problem-solving, and mentoring programs instead of training alone.^103^ As such, continuous training (i.e., preservice and on-the-job) and supportive supervision should be implemented for surveillance staff and focus on key competencies that include surveillance data management, analysis, and use across VPDs.

One critical requirement for a successful integrated VPD surveillance system is leadership convening stakeholders from different departments, including epidemiologists, microbiologists, and other key groups and sectors.^84^ If not designed to meet the needs of decision makers, integrated information systems can become burdensome and have detrimental effects on data quality and utility. As such, the design of surveillance information systems should be aligned with the required elements for use by decision makers, including considerations of unique identifiers to link epidemiological and laboratory surveillance data for individual cases.

The design of surveillance information systems should be aligned with the required elements for use by decision makers.

### Limitations

The literature search retrieved more than 5,000 references following a search strategy that included terms related to “integration,” whereas more than 20,000 references were retrieved without specifying these terms. Our use of the former search strategy may have limited the retrieval of articles describing innovations and best practices for disease-specific surveillance systems that might be applied to comprehensive VPD surveillance. Differential yields between published and gray literature for information on some of the support functions, especially program management and supervision, were observed. Publication bias may also have affected the robustness of our findings. However, our findings were supplemented with published and gray literature from VPD surveillance experts at WHO WPRO, the WHO Headquarters, and the U.S. Centers for Disease Control and Prevention. Gray literature provided important perspectives that may not be captured in published sources alone; however, some did not describe the methods used, and by nature, they were not peer reviewed. The screening and the analysis of the references on syndromic surveillance were done by 1 reviewer. Although extra checks were performed to improve the analysis and all results were discussed by the research team, some bias may have resulted. Additionally, our review methods were limited to a desk review of published and gray literature from 2000 to 2021. It would benefit from a review focused on the most recent literature on coronavirus disease (COVID-19) integration into existing surveillance systems as well as an in-depth, field-based review of program operations at the national and subnational levels.

## CONCLUSION

VPD surveillance can be strengthened in the WPR and resources can be optimized by bolstering the support functions and further integration of VPDs and other diseases as part of a comprehensive health systems approach. Opportunities exist for integration, such as in the functions of surveillance workforce, laboratory capacity, and information systems and data management. The integration of VPDs into broader communicable disease surveillance systems is further encouraged while ensuring that the minimal WHO-recommended standards for VPD surveillance are met. The development of legal frameworks, guidance documents, and coordination mechanisms are critical to enable complete and timely reporting and investigation of VPD cases and clusters. The outcomes of this review could inform WPR country implementation plans, following the endorsement of the Regional Strategic Framework for Vaccine-Preventable Diseases and Immunization in the Western Pacific 2021–2030.

## Supplementary Material

GHSP-D-22-00017-supplement.pdf

## Notes

**Peer Reviewed**
