# Supplementary material for: Comprehensive Vaccine-Preventable Disease Surveillance in the Western Pacific Region: A Literature Review on Integration of Surveillance Functions, 2000–2021
Source: Glob Health Sci Pract. 2022 Oct 31;10(5):e2200017. doi: 10.9745/GHSP-D-22-00017 (PMC9622275; doi:10.9745/GHSP-D-22-00017)
Supplement: GHSP-D-22-00017-supplement.pdf [file GHSP-D-22-00017-supplement.pdf]

Supplement to: Donadel M, Scobie HM, Pastore R, et al. Comprehensive vaccine-preventable disease surveillance in the Western Pacific Region: a literature review on integration of surveillance functions, 2000–2021. *Glob Health Sci Pract*. 2022;10(5):e2200017. https://doi.org/10.9745/GHSP-D-22-00017

Supplement Table S1: Search strategies developed for the literature review on comprehensive vaccine-preventable disease surveillance, by database and search date

**Database**	**Strategy**	**Records** 11/25/2019	**Records** 12/17/2020	**Records** 10/28/2021
**Medline (OVID) 1946-**	(Vaccine preventable ADJ2 disease*) OR (Vaccine preventable ADJ2 infection*) OR (Vaccine preventable ADJ2 illness*) OR VPD* OR cholera OR rubella OR diphtheria OR influenzae OR hepatitis OR human papillomavirus OR influenza OR encephalitis OR measles OR meningococc* OR mumps OR tetanus OR pertussis OR pneumococc* OR polio* OR rotavirus OR rubella OR typhoid OR varicella OR yellow fever<br><br>AND<br><br>(surveillance ADJ5 (integrat* OR expand* OR expansion OR comprehensive OR link* OR adapt* OR joint OR combine* OR platform))<br><br>Limit to 2000 - ; (201911* OR 201912* OR 2020* OR 2021*).dt (202012* OR 2021*).dt	735	78	73
**Embase (OVID) 1996-**	(Vaccine preventable ADJ2 disease*) OR (Vaccine preventable ADJ2 infection*) OR (Vaccine preventable ADJ2 illness*) OR VPD* OR cholera OR rubella OR diphtheria OR influenzae OR hepatitis OR human papillomavirus OR influenza OR encephalitis OR measles OR meningococc* OR mumps OR tetanus OR pertussis OR pneumococc* OR polio* OR rotavirus OR rubella OR typhoid OR varicella OR yellow fever<br><br>AND<br><br>(surveillance ADJ5 (integrat* OR expand* OR expansion OR comprehensive OR link* OR adapt* OR joint OR combine* OR platform))<br><br>Limit to 2000 - ; NOT pubmed/medline ; (201911* OR 201912* OR 2020* OR 2021*).dc	798<br><br>-484 duplicates<br><br>=314 unique items	138<br><br>-79 duplicates<br><br>=59 unique items	118<br><br>-54 duplicates<br><br>=64 unique items
**Global Health (OVID) 1973-**	(Vaccine preventable ADJ2 disease*) OR (Vaccine preventable ADJ2 infection*) OR (Vaccine preventable ADJ2 illness*) OR VPD* OR cholera OR rubella OR diphtheria	622<br><br>-416 duplicates	61<br><br>-43 duplicates	53<br><br>-27 duplicates

**Database**	**Strategy**	**Records** 11/25/2019	**Records** 12/17/2020	**Records** 10/28/2021
	OR influenzae OR hepatitis OR human papillomavirus OR influenza OR encephalitis OR measles OR meningococc* OR mumps OR tetanus OR pertussis OR pneumococc* OR polio* OR rotavirus OR rubella OR typhoid OR varicella OR yellow fever<br><br>AND<br><br>(surveillance ADJ5 (integrat* OR expand* OR expansion OR comprehensive OR link* OR adapt* OR joint OR combine* OR platform))<br><br>Limit to 2000 - ; (201911* OR 201912* OR 2020* OR 2021*).dc	=206 unique items	=18 unique items	=26 unique items
**CINAHL (Ebsco)**	(“Vaccine preventable” N2 disease*) OR (“Vaccine preventable” N2 infection*) OR (Vaccine preventable N2 illness*) OR VPD* OR cholera OR rubella OR diphtheria OR influenzae OR hepatitis OR “human papillomavirus” OR influenza OR encephalitis OR measles OR meningococcus OR mumps OR tetanus OR pertussis OR pneumococc* OR polio* OR rotavirus OR rubella OR typhoid OR varicella OR “yellow fever”<br><br>AND<br><br>(surveillance N5 (integrat* OR expand* OR expansion OR comprehensive OR link* OR adapt* OR joint OR combine* OR platform))<br><br>Limit to 2000 - ; exclude Medline records	25<br><br>-16 duplicates<br><br>=9 unique items	11<br><br>-10 duplicates<br><br>=1 unique items	8<br><br>-2 duplicates<br><br>=6 unique items
**Africa Wide Information (Ebsco)**	(“Vaccine preventable” N2 disease*) OR (“Vaccine preventable” N2 infection*) OR (Vaccine preventable N2 illness*) OR VPD* OR cholera OR rubella OR diphtheria OR influenzae OR hepatitis OR “human papillomavirus” OR influenza OR encephalitis OR measles OR meningococcus OR mumps OR tetanus OR pertussis OR pneumococc* OR polio* OR rotavirus OR rubella OR typhoid OR varicella OR “yellow fever”	585<br><br>-351 duplicates<br><br>=234 unique items	0	0

**Database**	**Strategy**	**Records** 11/25/2019	**Records** 12/17/2020	**Records** 10/28/2021
	AND<br><br>(surveillance N5 (integrat* OR expand* OR expansion OR comprehensive OR link* OR adapt* OR joint OR combine* OR platform))<br><br>Limit to 2000 - ;			
**Scopus**	TITLE-ABS-KEY((“Vaccine preventable” W/2 disease*) OR (“Vaccine preventable” W/2 infection*) OR (Vaccine preventable W/2 illness*) OR VPD* OR cholera OR rubella OR diphtheria OR influenzae OR hepatitis OR “human papillomavirus” OR influenza OR encephalitis OR measles OR meningococcus OR mumps OR tetanus OR pertussis OR pneumococc* OR polio* OR rotavirus OR rubella OR typhoid OR varicella OR “yellow fever”) AND TITLE-ABS-KEY((surveillance W/5 integrat*) OR (surveillance W/5 expand*) OR (surveillance W/5 expansion) OR (surveillance W/5 comprehensive) OR (surveillance W/5 link*) OR (surveillance W/5 adapt*) OR (surveillance W/5 joint) OR (surveillance W/5 combine*) OR (surveillance W/5 platform)) AND NOT INDEX(medline)<br><br>Limit to 2000 - ;	180<br><br>-124 duplicates<br><br>=56 unique items	13<br><br>-7 duplicates<br><br>=6 unique items	22<br><br>-14 duplicates<br><br>=8 unique items
**ProQuest Central**	(((integrat* NEAR/5 surveillance) OR (surveillance NEAR/5 expand*) OR (surveillance NEAR/5 expansion) OR (surveillance NEAR/5 comprehensive) OR (surveillance NEAR/5 link*) OR (surveillance NEAR/5 adapt*) OR (surveillance NEAR/5 joint) OR (surveillance NEAR/5 combine*) OR (surveillance NEAR/5 platform)) AND "vaccine preventable")<br><br>Abstract Available; Limit 2000 -	587<br><br>-117 duplicates<br><br>=470 unique items	191<br><br>-48 duplicates<br><br>=143 unique items	173<br><br>-36 duplicates<br><br>=137 unique items

**Database**	**Strategy**	**Records** 11/22/2019	**Records** 12/17/2020	**Records** 10/28/2021
**Medline (OVID) 1946-**	(syndromic surveillance OR disease surveillance OR (outbreak ADJ5 surveillance) OR (early warning ADJ5 surveillance) OR (global health security ADJ5 surveillance) OR (epidemic ADJ5 surveillance) OR (public health ADJ5 surveillance) OR (IDSR ADJ5 surveillance) OR (EWAR* ADJ5 surveillance) OR (GHS* ADJ5 surveillance)).ti OR (integrated disease surveillance and response).ti,ab.<br><br>AND<br><br>Review.pt<br><br>Limit 2000- ; (201911* OR 201912* OR 2020* OR 2021*).dt	186	12	16
**Embase (OVID) 1947-**	(syndromic surveillance OR disease surveillance OR (outbreak ADJ5 surveillance) OR (early warning ADJ5 surveillance) OR (global health security ADJ5 surveillance) OR (epidemic ADJ5 surveillance) OR (public health ADJ5 surveillance) OR (IDSR ADJ5 surveillance) OR (EWAR* ADJ5 surveillance) OR (GHS* ADJ5 surveillance)).ti OR (integrated disease surveillance and response).ti,ab.<br><br>AND<br><br>Review.pt<br><br>Limit 2000-; (201911* OR 201912* OR 2020* OR 2021*).dc	205<br><br>-118 duplicates<br><br>=87 unique items	27<br><br>-7 duplicates<br><br>=20 unique items	29<br><br>-14 duplicates<br><br>=15 unique items
**Global Health (OVID) 1973-**	(syndromic surveillance OR disease surveillance OR (outbreak ADJ5 surveillance) OR (early warning ADJ5 surveillance) OR (global health security ADJ5 surveillance) OR (epidemic ADJ5 surveillance) OR (public health ADJ5 surveillance) OR (IDSR ADJ5 surveillance) OR (EWAR* ADJ5 surveillance) OR (GHS* ADJ5 surveillance)).ti OR (integrated disease surveillance and response).ti,ab.<br><br>AND<br><br>Review.ti	40<br><br>-25 duplicates<br><br>=15 unique items	4<br><br>-2 duplicates<br><br>=2 unique items	9<br><br>-2 duplicates<br><br>= 7 unique items

**Database**	**Strategy**	**Records** 11/22/2019	**Records** 12/17/2020	**Records** 10/28/2021
	Limit 2000-; (201911* OR 201912* OR 2020* OR 2021*).up			
**CINAHL (Ebsco)**	(TI ("syndromic surveillance" OR "disease surveillance" OR (outbreak N5 surveillance) OR ("early warning" N5 surveillance) OR ("global health security" N5 surveillance) OR (epidemic N5 surveillance) OR ("public health" N5 surveillance)) OR ("integrated disease surveillance and response")) OR (AB ("syndromic surveillance" OR "disease surveillance" OR (outbreak N5 surveillance) OR ("early warning" N5 surveillance) OR ("global health security" N5 surveillance) OR (epidemic N5 surveillance) OR ("public health" N5 surveillance) OR (IDSR N5 surveillance) OR (EWAR* N5 surveillance) OR (GHS* N5 surveillance)) OR ("integrated disease surveillance and response"))<br><br>**Limiters** - Exclude MEDLINE records; Publication Type: Review; Limit 2000-	19<br><br>-1 duplicates<br><br>=18 unique items	27<br><br>-5 duplicates<br><br>=22 unique items	8<br><br>-0 duplicates<br><br>= 8 unique items
**Africa Wide Information**	(TI ("syndromic surveillance" OR "disease surveillance" OR (outbreak N5 surveillance) OR ("early warning" N5 surveillance) OR ("global health security" N5 surveillance) OR (epidemic N5 surveillance) OR ("public health" N5 surveillance)) OR ("integrated disease surveillance and response")) OR (AB ("syndromic surveillance" OR "disease surveillance" OR (outbreak N5 surveillance) OR ("early warning" N5 surveillance) OR ("global health security" N5 surveillance) OR (epidemic N5 surveillance) OR ("public health" N5 surveillance) OR (IDSR N5 surveillance) OR (EWAR* N5 surveillance) OR (GHS* N5 surveillance)) OR ("integrated disease surveillance and response"))<br><br>**Limiters** - Scholarly (Peer Reviewed) Journals ; Limit 2000-	0	0	0
**Scopus**	(TITLE("syndromic surveillance" OR "disease surveillance" OR (outbreak W/5 surveillance) OR ("early warning" W/5 surveillance) OR ("global health security" W/5 surveillance) OR (epidemic W/5 surveillance) OR ("public health" W/5 surveillance) OR (IDSR W/5 surveillance) OR	42<br><br>-7 duplicates<br><br>=35	6<br><br>-3 duplicates<br><br>=3	50<br><br>-2 duplicates<br><br>=48

**Database**	**Strategy**	**Records** 11/22/2019	**Records** 12/17/2020	**Records** 10/28/2021
	(EWAR* W/5 surveillance) OR (GHS* W/5 surveillance)) OR TITLE-ABS("integrated disease surveillance and response")) AND NOT INDEX(medline) AND NOT INDEX(embase)<br><br>Limit 2000-	unique items	unique items	unique items

Supplement to: Donadel M, Scobie HM, Pastore R, et al. Comprehensive vaccine-preventable disease surveillance in the Western Pacific Region: a literature review on integration of surveillance functions, 2000–2021. *Glob Health Sci Pract*. 2022;10(5):e2200017. https://doi.org/10.9745/GHSP-D-22-00017

Supplement Table S2: Countries referenced in the articles included (n=87) - literature review on comprehensive vaccine-preventable disease surveillance in the WHO Western Pacific Region, 2010-2021

Country	Number of articles in which the country is referenced
China	16
Vietnam	14
Cambodia	13
Lao PDR	13
Philippines	12
Fiji	9
Mongolia	9
Papua New Guinea	9
Australia	6
Japan	6
Malaysia	6
Chinese Taipei	5
Singapore	5
New Zealand	4
Republic of Korea	4
Solomon Islands	4
Hong Kong	3
Samoa	3
Tuvalu	3
Vanuatu	3
Brunei Darussalam	2
Federated States of Micronesia	2
French Polynesia	2
Macao	2
Marshall Islands	2
Nauru	2
New Caledonia	2
Northern Mariana Islands	2
Palau	2
Tonga	2
Wallis and Futuna	2
American Samoa	1
Cook Islands	1
Guam	1
Kiribati	1
Niue	1
Pitcairn island	1
Tokelau	1
Regional*	13

Global*	11

*Publications with aggregated regional or global data.

Supplement to: Donadel M, Scobie HM, Pastore R, et al. Comprehensive vaccine-preventable disease surveillance in the Western Pacific Region: a literature review on integration of surveillance functions, 2000–2021. *Glob Health Sci Pract*. 2022;10(5):e2200017. https://doi.org/10.9745/GHSP-D-22-00017

Supplement Table S3: Articles included in the review by selected characteristics (n=87) - literature review on comprehensive vaccine-preventable disease surveillance in the WHO Western Pacific Region, 2010-2021

Title	Type of publication	Place	Type(s) of surveillance	Surveillance support functions integrated	Enabling factors/ effectiveness of integration described
Craig et al. (2018)[11]	Published	Solomon Islands, LMIC	Case-based surveillance, nationwide implementation	Governance, Program management, Workforce capacity, Field logistics and communication, Data management and use, Coordination	☒Enabling factor ☒Effectiveness
Davgasuren et al. (2019)[12]	Published	Mongolia, LMIC	Case-based surveillance, nationwide implementation	Governance, Workforce capacity, Field logistics and communication, Data management and use, Coordination	☐Enabling factor ☐Effectiveness
Calain (2007)[13]	Published	Cambodia, Lao PDR, LMICs	Case-based surveillance, nationwide implementation	Workforce capacity	☒Enabling factor ☐Effectiveness
Chretien et al. (2008) [14]	Published	Global, multiple income -levels	Case-based surveillance, nationwide implementation	Workforce capacity, Field logistics and communication, Data management and use	☒Enabling factor ☐Effectiveness
May et al. (2009) [15]	Published	Chinese Taipei, Vanuatu, Solomon Islands, Samoa, multiple	Case-based surveillance, nationwide implementation	Workforce capacity, Field logistics and communication, Laboratory, Data management and use, Coordination	☐Enabling factor ☒Effectiveness

		income - levels			
Li et al. (2012)[16]	Published	China, UMIC	Case-based surveillance, nationwide implementation	Governance, Laboratory, Data management and use	☐Enabling factor ☒Effectiveness
Clara et al. (2020) [17]	Published	Vietnam, LMIC	Event-based surveillance, implementation in 6 provinces	Governance, Supervision	☒Enabling factor ☒Effectiveness
Craig et al. (2020) [18]	Published	Pacific island countries and territories, multiple income levels	Event-based, case-based surveillance, nationwide implementation	Data management and use	☐Enabling factor ☐Effectiveness
Jayatilleke et al. (2020) [19]	Published	Global, LMIC	Aggregate surveillance, nationwide implementation	Data management and use	☒Enabling factor ☐Effectiveness
Zurynski et al. (2013) [20]	Published	Australia, HIC	Case-based surveillance, nationwide implementation	Workforce capacity, Field Logistics and communication, Laboratory, Coordination	☒Enabling factor ☐Effectiveness
Touch et al. (2009) [21]	Published	Cambodia, LMIC	Case-based surveillance, nationwide implementation	Governance, Workforce capacity, Laboratory, Data management and use	☐Enabling factor ☒Effectiveness
Cambodia National Immunization Program (2015)[22]	Gray	Cambodia, LMIC	Case-based, aggregate surveillance, nationwide implementation	Program management, Workforce capacity, Field Logistics and communication, Supervision, Data management and use	☐Enabling factor ☐Effectiveness
Cambodia National Immunization Program (2017)[23]	Gray	Cambodia, LMIC	Case-based, nationwide implementation	Governance, Workforce capacity, Field Logistics and communication, Data management and use	☐Enabling factor ☐Effectiveness

Touch et al. (2009) [24]	Published	Cambodia, LMIC	Case-based, aggregate surveillance, nationwide implementation	Governance, Workforce capacity, Field Logistics and communication, Laboratory	☒Enabling factor ☒Effectiveness
Wang et al. (2008) [25]	Published	China, UMIC	Case-based, aggregate surveillance, nationwide implementation	Workforce capacity	☐Enabling factor ☐Effectiveness
World Health Organization. (2012)[26]	Published	China, UMIC	Case-based, aggregate surveillance, nationwide implementation	Governance, Program management, Workforce capacity, Field Logistics and communication, Laboratory, Data management and use	☒Enabling factor ☐Effectiveness
Cavallaro et al. (2015) [27]	Published	China, UMIC	Case-based, aggregate surveillance, nationwide implementation	Governance, Workforce capacity, Field Logistics and communication, Laboratory, Supervision	☒Enabling factor ☒Effectiveness
Sandhu (2013)[28]	Published	China, UMIC	Case-based, aggregate surveillance, nationwide implementation	Workforce capacity, Field Logistics and communication, Laboratory, Data management and use	☒Enabling factor ☐Effectiveness
World Health Organization, Western Pacific Regional Office. (2017)[29]	Gray	Fiji, UMIC	Case-based surveillance, single site implementation	Governance, Workforce capacity, Field Logistics and communication, Laboratory, Data management and use	☒Enabling factor ☐Effectiveness
Dunne et al. (2016) [30]	Published	Fiji, UMIC	Case-based surveillance, single site implementation	Governance, Field Logistics and communication, Laboratory	☒Enabling factor ☐Effectiveness

World Health Organization (2019) [31]	Gray	Fiji, UMIC	Case-based surveillance, single site implementation	Workforce capacity, Field Logistics and communication	☒Enabling factor ☐Effectiveness
Rota et al. (2011) [32]	Published	Global, multiple income levels	Case-based surveillance, nationwide implementation	Laboratory	☒Enabling factor ☒Effectiveness
World Health Organization, Western Pacific Regional Office (2017)[33]	Published	Global, multiple income levels	Case-based, aggregate, event-based surveillance, nationwide implementation	Governance, Workforce capacity, Data management and use, Coordination	☒Enabling factor ☐Effectiveness
World Health Organization (2000) [34]	Published	Global, multiple income levels	Case-based, aggregate surveillance, nationwide implementation	Governance, Workforce capacity, Field Logistics and communication, Laboratory	☐Enabling factor ☐Effectiveness
World Health Organization (2018) [35]	Gray	Global, multiple income levels	Case-based surveillance, sentinel sites	Laboratory	☐Enabling factor ☐Effectiveness
World Health Organization, Western Pacific Regional Office. (2019)[36]	Gray	Regional (WPR), multiple income levels	Case-based surveillance, nationwide implementation	Laboratory	☒Enabling factor ☐Effectiveness
World Health Organization, Western Pacific Regional Office. (2017)[7]	Gray	Regional (WPR), multiple income levels	Case-based, aggregate surveillance, nationwide implementation	Data management and use	☐Enabling factor ☐Effectiveness
World Health Organization (2014) [37]	Published	Bi-regional (WPR, SEAR), multiple income levels	Case-based surveillance, sentinel sites	Laboratory	☐Enabling factor ☐Effectiveness
World Health Organization (2019) [38]	Gray	Global, multiple income levels	Case-based surveillance, sentinel sites	Laboratory	☐Enabling factor ☒Effectiveness
Heffelfinger et al. (2017) [39]	Published	Bi-regional (WPR, SEAR), multiple income levels	Case-based surveillance, sentinel sites	Governance	☐Enabling factor ☐Effectiveness
Biellik et al. (2018) [40]	Published	Global, multiple income levels	Case-based surveillance,	Governance, Workforce capacity, Data	☐Enabling factor

			nationwide implementation	management and use	☐Effectiveness
Gavi the Vaccine Alliance (Gavi) (2019)[41]	Gray	Lao PDR, LMIC	Case-based surveillance, nationwide implementation	Workforce capacity, Data management and use	☐Enabling factor ☐Effectiveness
Lao People's Democratic Republic National Centre for Laboratory and Epidemiology. (2013) [42]	Gray	Lao PDR, LMIC	Case-based, aggregate, event-based surveillance, nationwide implementation	Governance, Workforce capacity, Supervision	☐Enabling factor: ☐Effectiveness
World Health Organization, UNICEF, GAVI Alliance, Luxembourg Agency for Development Cooperation, and U.S. Centers for Disease Control and Prevention. (2012) [43]	Gray	Lao PDR, LMIC	Case-based surveillance, Nationwide implementation	Workforce capacity	☐Enabling factor ☐Effectiveness
Lao People's Democratic Republic Ministry of Health, World Health Organization, and U.S. Centers for Disease Control and Prevention. (2015) [44]	Gray	Lao PDR, LMIC	Case-based, event-based surveillance, nationwide implementation	Governance, Program management, Workforce capacity, Field Logistics and communication, Laboratory, Supervision, Data management and use, Coordination	☐Enabling factor ☐Effectiveness
Lao People's Democratic Republic Ministry of Health, World Health Organization, and U.S. Centers for	Gray	Lao PDR, LMIC	Case-based, aggregate, event-based surveillance, Regional implementation	Governance, Workforce capacity, Field Logistics and communication, Laboratory, Supervision, Coordination	☐Enabling factor ☐Effectiveness

Disease Control and Prevention. (2017)[45]					
World Health Organization, Western Pacific Regional Office. (2019)[46]	Gray	Lao PDR, LMIC	Case-based, aggregate surveillance, nationwide implementation	Data management and use	☐Enabling factor ☐Effectiveness
World Health Organization, Western Pacific Regional Office. (2015)[47]	Gray	Mongolia, LMIC	Case-based surveillance, capital city implementation	Governance, Workforce capacity, Field Logistics and communication, Laboratory	☐Enabling factor ☐Effectiveness
World Health Organization, Western Pacific Regional Office. (2016)[48]	Gray	Mongolia, LMIC	Case-based surveillance, nationwide implementation	Workforce capacity, Field Logistics and communication, Laboratory	☒Enabling factor ☐Effectiveness
Mongolia Ministry of Health, World Health Organization, UNICEF, and U.S. Centers for Disease Control and Prevention. (2017)[49]	Gray	Mongolia, LMIC	Case-based surveillance, nationwide implementation	Governance	☐Enabling factor ☐Effectiveness
World Health Organization, Western Pacific Regional Office. (2015)[50]	Gray	Philippines, LMIC	Case-based surveillance, nationwide implementation	Field Logistics and communication, Laboratory	☒Enabling factor ☐Effectiveness
Republic of the Philippines Department of Health, World Health Organization, and U.S. Centers for Disease Control and Prevention. (2016)[51]	Gray	Philippines, LMIC	Case-based, event-based surveillance, nationwide implementation	Governance, Workforce capacity, Field Logistics and communication, Laboratory, Supervision, Data management and use	☒Enabling factor ☒Effectiveness

World Health Organization. (2019)[52]	Gray	Philippines, LMIC	Case-based, event-based surveillance, nationwide implementation	Laboratory	☐Enabling factor ☐Effectiveness
World Health Organization, Western Pacific Regional Office. (2019)[53]	Gray	Philippines, LMIC	Case-based, event-based surveillance, nationwide implementation	Governance	☐Enabling factor ☐Effectiveness
World Health Organization, Western Pacific Regional Office. (2016)[54]	Gray	PNG, LMIC	Case-based surveillance, implementation in major cities (Port Moresby, Lae)	Laboratory	☒Enabling factor ☐Effectiveness
World Health Organization, Western Pacific Regional Office. (2016)[55]	Gray	PNG, LMIC	Case-based, aggregate surveillance, national implementation	Governance, Workforce capacity, Laboratory	☒Enabling factor ☐Effectiveness
Papua New Guinea National Department of Health, World Health Organization, and U.S. Centers for Disease Control and Prevention. (2017)[56]	Gray	PNG, LMIC	Case-based surveillance, nationwide implementation	Governance, Program management	☐Enabling factor ☐Effectiveness
World Health Organization, Western Pacific Regional Office. (2019)[57]	Gray	PNG, LMIC	Case-based surveillance, nationwide implementation	Governance, Program management, Workforce capacity, Field Logistics and communication, Laboratory, Data management and use, Coordination	☒Enabling factor ☒Effectiveness
World Health Organization, Western Pacific Regional Office. (2019)[58]	Gray	Western Pacific Region, multiple income levels	Case-based surveillance, nationwide implementation	Data management and use	☒Enabling factor ☐Effectiveness

World Health Organization, Western Pacific Regional Office. (2018)[59]	Gray	Western Pacific Region, multiple income levels	Case-based surveillance, nationwide implementation	Laboratory	☒Enabling factor ☐Effectiveness
World Health Organization, Western Pacific Regional Office. (2018)[60]	Gray	Western Pacific Region, multiple income levels	Case-based surveillance, nationwide implementation	Governance, Laboratory, Supervision, Data management and use	☒Enabling factor ☐Effectiveness
World Health Organization, Western Pacific Regional Office. (2013)[61]	Published	Western Pacific Region, multiple income levels	Case-based surveillance, nationwide implementation	Governance, Workforce capacity, Field Logistics and communication, Laboratory, Data management and use	☐Enabling factor ☐Effectiveness
World Health Organization, Western Pacific Regional Office. (2020)[62]	Gray	Western Pacific Region, multiple income levels	Case-based, aggregate, event-based surveillance, nationwide implementation	Governance, Program management, Workforce capacity, Data management and use	☐Enabling factor ☐Effectiveness
World Health Organization, Western Pacific Regional Office. (2019)[63]	Gray	Western Pacific Region, multiple income levels	Case-based, aggregate surveillance, nationwide implementation	Field Logistics and communication, Data management and use	☒Enabling factor ☐Effectiveness
World Health Organization, Western Pacific Regional Office. (2019)[64]	Gray	Western Pacific Region, multiple income levels	Case-based, aggregate surveillance, nationwide implementation	Field Logistics and communication, Laboratory	☒Enabling factor ☐Effectiveness
World Health Organization, Western Pacific Regional Office. (2019)[65]	Gray	Western Pacific Region, multiple income levels	Case-based, aggregate surveillance, nationwide implementation	Workforce capacity, Field logistics and communication, Laboratory	☐Enabling factor ☐Effectiveness
World Health Organization, Western Pacific Regional Office. (2019)[66]	Gray	Western Pacific Region, multiple income levels	Case-based, aggregate surveillance, nationwide implementation	Governance, Field Logistics and communication, Laboratory, Data management and use	☒Enabling factor ☒Effectiveness

World Health Organization, Western Pacific Regional Office. (2013)[67]	Gray	Australia, Brunei Darussalam, Cambodia, China, Fiji, Hong Kong, Japan, Lao PDR, Malaysia, Mongolia, New Zealand, PNG, Philippines, Republic of Korea, Singapore, Vietnam, multiple income levels	Case-based surveillance, nationwide implementation	Laboratory	☐Enabling factor<br>☒Effectiveness
World Health Organization, Western Pacific Regional Office. (2019)[68]	Gray	Australia, Brunei Darussalam, Hong Kong, Macao, Japan, Malaysia, New Zealand, Philippines, PNG, South Korea, Singapore, Pacific Island Countries, Vietnam, Lao PDR, Mongolia, China, Cambodia, multiple income levels	Case-based surveillance, nationwide implementation	Field Logistics and communication, Data management and use	☐Enabling factor<br>☐Effectiveness
World Health Organization, Western Pacific Regional Office. (2019)[69]	Gray	Cambodia, Lao PDR, Mongolia, Vietnam, Fiji, Philippines, Cambodia, Macao, Hong Kong, Australia, PNG, Japan,	Case-based surveillance, nationwide implementation	Data management and use	☐Enabling factor<br>☐Effectiveness

		multiple income levels			
World Health Organization, Western Pacific Regional Office. (2015)[70]	Gray	China, Mongolia, Vietnam, Philippines, Fiji, Cambodia, Lao PDR, PNG, multiple income levels	Case-based surveillance, nationwide implementation	Governance, Field Logistics and communication	☒Enabling factor ☐Effectiveness
World Health Organization, Western Pacific Regional Office. (2011)[71]	Gray	Mongolia, PNG, Philippines, Vietnam, multiple income levels	Case-based surveillance, nationwide implementation	Governance, Program management, Field Logistics and communication, Laboratory, Data management and use	☒Enabling factor ☐Effectiveness
World Health Organization, Western Pacific Regional Office. (2018)[72]	Gray	Western Pacific Region, multiple income levels	Case-based surveillance, nationwide implementation	Program management, Workforce capacity, Field Logistics and communication, Data management and use, Coordination	☐Enabling factor ☐Effectiveness
World Health Organization, Western Pacific Regional Office. (2019)[73]	Gray	Western Pacific Region, multiple income levels	Case-based surveillance, nationwide implementation	Laboratory	☒Enabling factor ☐Effectiveness
White et al. (2017) [74]	Published	Samoa, UMIC	Case-based surveillance, nationwide implementation	Workforce capacity, Field logistics and communication, Laboratory, Data management and use	☐Enabling factor ☐Effectiveness
Chua et al. (2015) [75]	Published	Singapore, HIC	Case-based surveillance, nationwide implementation	Workforce capacity, Laboratory, Data management and use	☐Enabling factor ☒Effectiveness

Lau et al. (2018) [76]	Published	Singapore, HIC	Case-based surveillance, single-site implementation	Workforce capacity, Laboratory	☐Enabling factor ☐Effectiveness
Jian et al. (2017) [77]	Published	Chinese Taipei, HIC	Case-based, event-based surveillance, nationwide implementation	Governance, Workforce capacity, Data management and use	☐Enabling factor ☐Effectiveness
Nelesone et al. (2006) [78]	Published	Tuvalu, UMIC	Case-based surveillance, nationwide implementation	Governance, Workforce capacity, Field logistics and communication, Data management and use	☐Enabling factor ☒Effectiveness
Alroy et al. (2018) [79]	Published	Vietnam, LMIC	Case-based surveillance, national implementation	Governance, Workforce capacity, Laboratory, Data management and use	☒Enabling factor ☒Effectiveness
Vietnam National Institute of Hygiene and Epidemiology, World Health Organization, UNICEF, and U.S. Centers for Disease Control and Prevention. (2017) [80]	Gray	Vietnam, LMIC	Case-based surveillance, nationwide implementation	Governance, Workforce capacity, Supervision, Coordination	☐Enabling factor ☐Effectiveness
World Health Organization, Western Pacific Regional Office. (2019)[81]	Published	Vietnam, LMIC	Case-based surveillance, nationwide implementation	Workforce capacity, Data management and use	☒Enabling factor ☒Effectiveness
World Health Organization, Western Pacific Regional Office. (2015)[82]	Gray	Vietnam, LMIC	Case-based surveillance, nationwide implementation	Field logistics and communication, Laboratory	☐Enabling factor ☐Effectiveness
Taniguchi et al. (2007) [83]	Published	Japan, HIC	Case-based surveillance,	Governance, Workforce capacity, Field	☐Enabling factor ☐Effectiveness

			nationwide implementation	logistics and communication	
Hyde et al. (2013) [84]	Published	Global, multiple income - levels	Case-based surveillance, nationwide implementation	Governance	☒Enabling factor ☒Effectiveness
Phalkey et al. (2017) [85]	Published	Philippines, Vietnam, Malaysia, China, multiple income levels	Case-based surveillance, nationwide implementation	Governance	☐Enabling factor ☐Effectiveness
Shu et al. (2019) [86]	Published	China, UMIC	Case-based surveillance, nationwide implementation	Governance, Program management, Workforce capacity, Field logistics and communication, Laboratory, Data management and use, Coordination	☒Enabling factor ☐Effectiveness
Anh et al. (2020) [87]	Published	Vietnam, LMIC	Aggregate surveillance, nationwide implementation	Governance, Laboratory	☒Enabling factor ☐Effectiveness
Hsih et al. (2020) [88]	Published	Chinese Taipei, HIC	Case-based surveillance, nationwide implementation	Laboratory	☐Enabling factor ☐Effectiveness
Ma et al (2019)[89]	Published	China, UMIC	Case-based surveillance, nationwide implementation	Data management and use	☐Enabling factor ☐Effectiveness
El Guerche-Séblain et al (2021) [90]	Published	Australia, China, Malaysia, multiple income levels	Case-based surveillance, nationwide implementation	Governance, Workforce capacity, Field logistics and communication, Laboratory, Data management and use, Coordination	☐Enabling factor ☐Effectiveness
Qin et al (2010)[91]	Published	China, UMIC	Case-based surveillance, single facility	Laboratory	☐Enabling factor ☐Effectiveness
Manalili et al (2020) [92]	Published	Philippines, LMIC	Case-based surveillance,	Governance, Workforce	☐Enabling factor

			nationwide implementation	capacity, Field logistics and communication, Laboratory, Data management and use	☐Effectiveness
Aye et al (2020) [93]	Published	Malaysia, Cambodia, South Korea, Australia, New Zealand, Japan, China, Philippines, Singapore, Vietnam, Chinese Taipei, multiple income levels	Case-based surveillance, nationwide implementation	Governance, Program management, Workforce capacity, Laboratory	☐Enabling factor ☐Effectiveness
Quinn et al (2021) [94]	Published	China, Fiji, Chinese Taipei, multiple income levels	Case-based surveillance, nationwide implementation	Field logistics and communication	☐Enabling factor ☐Effectiveness
Gonzalez-Silva et al (2021) [95]	Published	Global, multiple income levels	Case-based surveillance, nationwide implementation	Governance, Workforce capacity, Field logistics and communication, Laboratory, Data management and use, Coordination	☐Enabling factor ☐Effectiveness
Chan et al (2021) [96]	Published	Cambodia, Laos, Vietnam, LMIC	Case-based surveillance, nationwide implementation	Laboratory	☐Enabling factor ☐Effectiveness

Definitions: LIC, Low Income Country; LMIC, Lower-Middle Income Country; UMIC, Upper-Middle Income Country; HIC, High Income Country; PNG: Papua New Guinea; US-CDC: U.S. Centers for Disease Control and Prevention; WHO: World Health Organization; WPRO: Western Pacific Regional Office
